# Opinions Differ

**Published:** 1879-08

**Authors:** 


OPINIONS DIFFER.—Dr. L. Duncan Bulkley, the eminent Dermatologist, thinks the Health Food Company's Whole Wheat Flour the best food extant. Dr. John Douglass, Dr. Mattson, and a thousand other Doctors, and ten thousand laymen, characterize the Company's WHITE WHEAT GLUTEN as the best food known to science; Dr. Jennings, and multitudes besides, declare the UNIVERSAL FOOD to be the one thing needed for infants, and invalids, and all sorts of feeble folks. Cyrus Bradbury, of Hopedale, Dr. Hanaford, of Reading, Mass., and hundreds besides, declare, that as constipation is well-nigh universal among Americans, and as the GLUTEN SUPPOSITORIES always cure that trouble, the aforesaid Suppositories must necessarily be the best article made by the Company. Dr. Dunbar says: "Abandon all besides, and devote your energies to the manufacture of the EXTRACT OF GLUTEN AND BARLEY, which is so effectual in Consumption, Scrofula, Paralysis, and all wasting diseases." Dr. Stone says: "Drop all the rest and supply the world with your GLUTEN SOAP, so that humanity shall be beautiful as well as healthful." Prof. Austin Flint cordially commends the Company's GLUTEN FLOUR, and the bread made from it, to all Diabetics. And so it goes, all around; there is not an article manufactured by the Health Food Co. which is not considered by somebody *the best thing in the world.* More than 100 letters have been received and published by the NEW YORK DAILY WITNESS since the first day of December, 1878, from doctors, clergymen, and other intelligent persons, all bearing earnest testimony to the great value of the Health Food Company's products in various diseases. This goes to show that each article is invaluable in its place. No one food will meet every case which presents itself. Foods must differ quite as much as diseases. Carbonaceous substances, in a condition to be very largely digested, may be just the thing for the consumptive, and yet may be of small value for the atheromic—the sufferer from fatty degeneration of the heart and other tissues. The sufferer who consults the highest authority, and is made whole by a given food, naturally thinks *that* the best for every other sick person. A careful perusal of all the printed matter published by the Health Food Co. will enlighten the reader upon all these points, and enable him to select intelligently.

